# West Nile virus in the Iberian Peninsula: using equine cases to identify high-risk areas for humans

**DOI:** 10.2807/1560-7917.ES.2023.28.40.2200844

**Published:** 2023-10-05

**Authors:** José-María García-Carrasco, Antonio-Román Muñoz, Jesús Olivero, Marina Segura, Ignacio García-Bocanegra, Raimundo Real

**Affiliations:** 1 Biogeography, Diversity and Conservation Lab, Department of Animal Biology, Faculty of Sciences, University of Málaga, Málaga, Spain; 2 International Vaccination Center of Malaga, Maritime Port of Malaga, Ministry of Health, Consumption and Social Welfare, Government of Spain, Málaga, Spain; 3 Departamento de Sanidad Animal, Facultad de Veterinaria, Universidad de Córdoba, Córdoba, Spain

**Keywords:** Distribution models, Favourability, Horse, One Health, Pathogeography, Prediction, Risk map, West Nile virus, Zoonoses

## Abstract

**Background:**

West Nile virus (WNV) is a flavivirus with an enzootic cycle between birds and mosquitoes; humans and horses are incidental dead-end hosts. In 2020, the largest outbreak of West Nile virus infection in the Iberian Peninsula occurred, with 141 clusters in horses and 77 human cases.

**Aim:**

We analysed which drivers influence spillover from the cycle to humans and equines and identified areas at risk for WNV transmission.

**Methods:**

Based on data on WNV cases in horses and humans in 2020 in Portugal and Spain, we developed logistic regression models using environmental and anthropic variables to highlight risk areas. Models were adapted to a high-resolution risk map.

**Results:**

Cases of WNV in horses could be used as indicators of viral activity and thus predict cases in humans. The risk map of horses was able to define high-risk areas for previous cases in humans and equines in Portugal and Spain, as well as predict human and horse cases in the transmission seasons of 2021 and 2022. We found that the spatial patterns of the favourable areas for outbreaks correspond to the main hydrographic basins of the Iberian Peninsula, jointly affecting Portugal and Spain.

**Conclusion:**

A risk map highlighting the risk areas for potential future cases could be cost-effective as a means of promoting preventive measures to decrease incidence of WNV infection in Europe, based on a One Health surveillance approach.

**Key public health message**

**What did you want to address in this study?**

West Nile fever is a viral infection transmitted to humans and other animals via mosquitoes. Infections of West Nile virus are increasing in Europe and the disease has a considerable impact on human and animal health. We wished to explore the environmental conditions that influence the transmission of the virus to humans and horses and test the role of horses as indicators of the disease in humans.

**What have we learnt from this study?**

Data on West Nile virus infection in horses can be useful indicators of risks of human cases. In addition, river basins play an important role as places where outbreaks occur and spread.

**What are the implications of your findings for public health?**

Horses can be used as part of an integrated surveillance system focused on reducing the number of people and animals infected with the virus. Decision-making would be more efficient using river basins as management units, instead of political administrative units, for example to alert the health facilities after infected horses have been detected.

## Introduction

West Nile virus (WNV) is a mosquito-borne arbovirus of the family Flaviviridae [1]. The virus is mainly transmitted from birds to mammals by blood-feeding ornithophilic mosquito species. Humans and equines are the main dead-end hosts: in humans, the virus may cause symptoms from febrile illness to neuroinvasive disease, although the latter occurs in less than 1% of cases [2]. Although the virus has been circulating in Europe since 1950, it was not until 2004 that Spain detected the first human case of West Nile neuroinvasive disease [3]. The same year, Portugal confirmed two cases of WNV infection in humans [4]. In 2010, Europe experienced the first large outbreak with 391 cases, and the same year, Spain had its first outbreak among equines and humans [5,6] and Portugal confirmed one human and two equine cases [7]. From 2011 to 2019, WNV activity was low but endemic in the Iberian Peninsula, with few outbreaks in equids and humans reported [5,8,9]. In the Iberian Peninsula, the largest outbreak to date occurred in 2020, affecting new regions. A total of 77 human cases were detected, including eight deaths [8,10]. Moreover, 141 clusters of WNV infection in horses occurred in the peninsula [9].

Equines are particularly sensitive to WNV infection. In horses, neurological disorders are more common than in humans [11]. Neurological symptoms of WNV infection in equines may include recumbency, cranial nerve deficits, muscular tremors, ataxia, hyperesthesia, fasciculations, convulsions, paralysis of the limbs, photophobia, vacuum chewing, disorientation, behavioural changes and tetany [12-15]. As WNV infection in equids is often symptomatic, horses, donkeys and mules could be useful identifiers of risk of virus circulation and the possibility of human infections and disease in areas with cases in equids [16]. Also, bird species of the family Corvidae can be used as sentinels for WNV detection, as the infection may result in high mortality in birds [17,18]. Indicator species for risk could be useful if there is a time window to implement control programmes to protect surrounding human communities.

Based on data of 77 human cases and 141 clusters in horses notified in the 2020 season, we aimed to analyse the geographical distribution of cases of WNV infection in horses and humans and create a risk map of the Iberian Peninsula to visualise environmental and spatial patterns from a macroecological perspective. We wanted to test if equine WNV infection cases could predict risk areas for human cases, which could be useful to implement early warning mechanisms, improve planning of surveillance measures and develop prevention policies in at-risk areas before outbreaks occur.

## Methods

### Data source and setting

Spain and Portugal report cases of WNV infection in humans to the European Surveillance System (TESSy) operated by the European Centre for Disease Prevention and Control (ECDC). We listed all areas in Spain and Portugal where cases of WNV infection in horses and humans occurred during the transmission season of 2020. We used cases that were detected during passive and active surveillance: clinical cases that were serologically positive (IgM) or with the virus identified using reverse transcription (RT) [19]. For data on humans, we used the 2020 data in TESSy [20]. In Europe, case locations are reported at the NUTS (Nomenclature of Territorial Units for Statistics) level 3 [21]. However, to achieve the highest possible accuracy of the geographical location of the cases, we compared the EU data with the data collected by the National Epidemiological Surveillance Network and the Coordinating Centre of Health Alerts and Emergencies (CCAES) [22,23]. Information on findings of WNV in horses in Spain and Portugal were obtained from the Veterinary Health Alert Risk system (RASVE), developed by the Spanish Ministry of Agriculture, Fisheries and Food. This system integrates health data from national and international sources, such as the Animal Disease Information System (ADIS) [24]. The outbreak data were retrieved in February 2022 and location of the outbreaks, in humans and horses, was georeferenced at the municipal level in Spain and at the parish level in Portugal. Municipalities had an average surface area of approximately 60 km^2^, while the average surface area of parishes was approximately 20 km^2^. For the modelling, municipalities and parishes were considered the operational geographic units (OGUs) in the Iberian Peninsula.

### Risk models

We elaborated separate risk models for cases of WNV infection in horses and in humans. First, using univariate score tests for the presence or absence of cases of WNV infection, we assessed the capacity of different environmental variables to explain the distribution of cases. Variables were selected on the basis of their potential predictive power and were assumed to be at least correlated with more proximal causal factors. Mosquito data were not available, therefore we used water availability and temperature as proxies of mosquito presence. The variables used comprised different factors, such as density of the human and animal population, distance to roads, production of crops, forests, river, altitude and precipitation. A list of all factors considered can be seen in the Supplementary table.

We controlled multicollinearity among variables by calculating pairwise Spearman correlation coefficients. If two variables belonging to the same factor were correlated by more than 0.8, the least explanatory variable, according to the score tests, was deleted. Moreover, we controlled the false discovery rate to avoid an increase in type I errors due to the number of variables used in the analysis [25]. Therefore, we organised the variables by importance and arranged them in descending order to better understand their influence on the occurrence of WNV infection. To determine their significance, we relied on the Rao score test [26]. To be included in subsequent steps, a variable must have a score-test probability below i*q/V, where i represents the variable's position in the order, q is the false discovery rate of 0.05, and V is the total number of variables. Consequently, we performed a multivariate forward stepwise logistic regression in which a variable was added to the null model if the resulting regression was most significantly improved by the new variable. A machine-learning algorithm, using maximum likelihood estimation, established the values of the parameters for the logistic regression. The result was a probability value of cases of WNV infection in each OGU according to its environmental and anthropic characteristics. The probability value of each OGU was transformed into a favourability value (F) using the favourability function [27]. The favourability value (ranging from 0 to 1) was calculated for each OGU, which represents the degree to which environmental conditions at that OGU favour the occurrence of WNV infection. The favourability model shows how the local probability of WNV cases differs from that expected by chance in the Iberian Peninsula and thus identifies localities with environmental conditions that favour infection with the virus. This was used for the elaboration of a risk map for cases of WNV infection in horses and humans.

### Relationship between cases in horses and humans

We evaluated the classification and discrimination capacity of the horse and human risk model. The classification power of the models, using a value of F = 0.5 as classification threshold, was estimated using the sensitivity, specificity, Cohen’s kappa and correct classification rate (CCR) [28] and the over- and under-prediction rates [29]. In contrast, the discrimination power was assessed using the area under the receiver operating characteristic curve (AUC) [30]. As we also wanted to test whether there was a relationship between cases in horses and humans, we tested each model with the alternative cases, that is, the horse model was evaluated with respect to the distribution of human cases, and the human model was evaluated with the distribution of cases in horses. In this way, we could test how useful it would be to use the distribution of cases in horses to predict risk for humans, and vice versa.

### Downscaling the risk models

To increase the potential utility of the cartographic model, we downscaled the model from its original OGU (municipalities or parishes) to 4 km^2^ squares using a direct downscaling approach [31]. We used the logistic regression equation to get probability values for 4 km^2^ squares according to the variable values for the same spatial resolution. In this way, we elaborated a risk map covering the entire Iberian Peninsula uniformly and at a high spatial resolution. Spatial analyses and the map display were elaborated using the geographic information systems QGIS 3.4 (www.qgis.org) and ArcGIS Desktop 10.7 (www.desktop.arcgis.com). Statistical analyses were performed using SPSS Statistics 26 (https://www.ibm.com).

## Results

The occurrence of cases in horses and humans was affected by several factors (Table 1). Temperature and precipitation played an important role in the distribution of cases in horses and humans. The mean annual temperature in areas with cases in horses was 17.25 ± 0.92°C, while it was 17.52 ± 0.64°C in areas with human cases. Cases in horses were affected by a positive precipitation seasonality (64.01 ± 10.28) and mean temperature (25.53 ± 1.13°C) of the hottest month, while human cases were positively correlated by mean annual precipitation (587.03 ± 111.02 mm) and solar irradiation (5.2 ± 0.09 W/m ^2^). Agricultural land use also influenced the localisation of outbreaks. Infections in horses were more common in areas with rice fields and irrigated land, such as permanently irrigated land, fruit trees and pastures, while human cases were more common in areas with irrigation systems.

**Table 1:** Explanatory variables included in the risk model of West Nile virus infection in horses and humans, Iberian Peninsula

Characteristics	Horses				Humans		
	B	Wald	Significance	B		Wald	Significance
Climatic factors							
Mean annual temperature	0.666	9.90	0.00165	0.831		6.80	0.00914
Mean temperature of the hottest month	0.284	6.14	0.0132	NA		NA	NA
Coefficient variation precipitation	0.107	33.15	< 0.0001	NA		NA	NA
Mean annual precipitation	NA	NA	NA	0.009		30.85	< 0.0001
Surface incoming solar radiation	NA	NA	NA	15.22		30.66	< 0.0001
Agricultural factors							
Mix of agricultural land with natural vegetation	-9.82	3.96	0.0466	NA		NA	NA
Rice fields	3.19	4.47	0.0344	NA		NA	NA
Irrigated land	1.41	6.40	0.0114	NA		NA	NA
Percentage of areas equipped with irrigation systems	NA	NA	NA	0.06		26.09	< 0.0001
Constant	-28.514	100.342	< 0.0001	-102.981		52.282	< 0.0001

NA: not applicable.

B is the coefficient that multiplies the variable values in the logit of the multivariate logistic regression.

The Wald parameter quantifies the relevance of the variable in the model.

Risk areas for WNV infection in humans were concentrated in the southern part of the Iberian Peninsula: along the coast, throughout the Guadalquivir River Basin and in two patches inland along the rivers Tagus and Guadiana (Figure). The high-risk areas for cases in horses were similar to those found in humans; however, the areas at risk were more extensive, with a wide zone in the south-western quadrant. The risk areas in the horse model also expanded northwards along the Mediterranean coast and extended inland following the river Ebro in the north-east of the peninsula (Figure).

**Figure:**
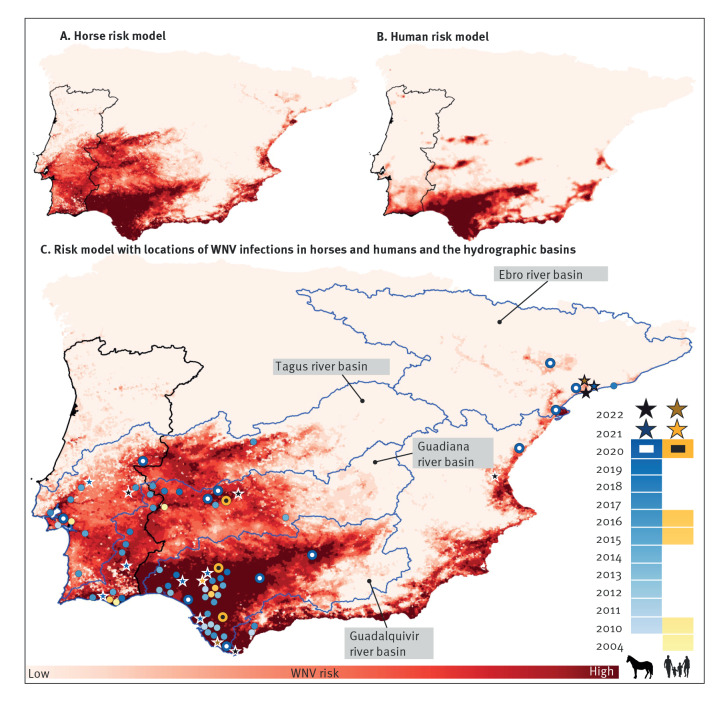
Risk areas for cases of West Nile virus infection in horses and humans, Iberian Peninsula

NUTS: Nomenclature of Territorial Units for Statistics; WNV: West Nile virus.

Notified cases of WNV infection in horses in blue and humans in yellow in the Iberian Peninsula at NUTS3. Bigger symbols with a white (horses) and black (humans) centre indicate cases in 2020. Star symbols indicate outbreaks in 2021 and 2022. The boundaries between Portugal and Spain are delineated by a black contour line.

Cartographically, risk models of horses and humans were very similar, although the high-risk areas for horses were more widespread. The risk areas identified by the horse model highlighted the main river basins in the Iberian Peninsula (Figure). All human cases of 2020 and all previous recorded outbreaks occurred in areas that were identified as high-risk by the horse model.

We evaluated the classification and discrimination capacity of the models. Both models showed high sensitivity, correctly detecting the cases in horses (sensitivity (Se) = 0.87) and humans (Se = 1). Equally, both models identified with high precision the absence of cases, showing a high specificity: 0.88 for horses and 0.95 for humans. Discrimination of the models was outstanding: 0.96 in horses and 0.99 in humans (Table 2). The horse model was evaluated with human cases, and the human model evaluated with the horse cases, which provided information about the utility of using cases in horses to predict risk for cases in humans, and vice versa. Both models showed a high sensitivity (> 0.81) and specificity (> 0.88). It is noteworthy that the horse model correctly classified all WNV cases in humans (Se = 1) and discriminated the human cases as effectively as the human model (Table 2).

**Table 2:** Comparative assessment of the classification and discrimination capacities of risk models of West Nile virus infection in horses and humans, Iberian Peninsula

Assessment	Horses		Humans	
	Horse data	Human data	Human data	Horse data
Kappa	0.06	0.03	0.07	0.13
Sensitivity	0.87	1	1	0.81
Specificity	0.88	0.88	0.95	0.95
CCR	0.88	0.88	0.95	0.95
Underprediction	0	0	0	0
Overprediction	0.96	0.98	0.96	0.92
AUC	0.96	0.99	0.99	0.95

AUC: area under the receiver operating characteristic curve; CCR: correct classification rate.

Data on notified cases in horses and humans in Spain and Portugal in 2020 were used. We used a favourability value of 0.5 as a cut-off point for classification purposes.

## Discussion

During the last decades, the situation with WNV infection in Europe has progressed from sporadic cases to yearly outbreaks that affect both equines and humans [9,32,33]. The number of notified cases in humans has increased [34] and seroprevalence in animals in Spain has increased from < 10% to > 20% in the last years [35,36]. Understanding the environmental factors that contribute to the spread of WNV is crucial since currently a vaccine is only available for horses but not for humans [37]. The virus is transmitted through mosquitoes and certain environmental factors can increase the likelihood of transmission to equines and humans. Some environmental variables that contribute to outbreaks are high temperatures, water availability and type of agricultural areas. High temperatures increase mosquito density [38-40] and the replication [41] and transmission [13,42,43] of the virus, while precipitation facilitates mosquito reproduction [44]. Croplands [45,46], particularly paddy fields, are ideal breeding sites for mosquitoes and can increase contact rates between hosts and vectors [47,48]. Areas that meet these conditions are favourable for spillovers and can affect equines and humans.

The horse model could be used to predict high-risk areas for equine and human cases. Evaluation of the models showed that the horse model could correctly classify risk areas for WNV infection in humans. This means that equids can be used as identifiers of risk areas where the virus could occur. Equids are normally more exposed to mosquito bites than humans due to their management conditions. Therefore, if an area experiences a spillover of WNV from the enzootic cycle, equines may be the first exposed. In humans, WNV infection is usually asymptomatic and there are no specific signs; cases may go unnoticed if neurological symptoms are not seen. Diagnosed cases in humans are only the tip of the iceberg: 80% of individuals infected with WNV are estimated to be asymptomatic and less than 1% develop a severe form of the disease. In that case, WNV infection may be a life-threatening condition, leading to serious complications in people over 50 years and in immunocompromised patients [49]. However, clinical disease is more evident in equines than in humans. This susceptibility to the virus makes equids a useful WNV indicator species for the human population, especially during the transmission season, when mosquitoes are abundant, and spillovers take place.

In Europe, a relationship between the occurrence of cases in equines and in humans has been observed [33]. In other countries, such as Italy and Greece, cases have been reported in horses in areas where there have been no notified cases in humans [50]. Additionally, in southern Spain, multiple areas of Andalusia have reported cases in equines where human cases have not yet been detected. Moreover, in the north-eastern part of Spain (Catalonia), our horse model has identified risk areas, whereas the human model has not, suggesting that spillovers affecting equines may also affect humans but remain undetected. In fact, the first confirmed human case in Catalonia occurred during the summer of 2022 in an area identified by our horse model, confirming equines as possible indicators of WNV [51]. In 2018, WNV was detected for the first time in birds and horses in Germany [52]. One year later, WNV was detected for the first time in humans in the same region and adjacent regions where it had been detected in horses the previous year. Areas with cases only in horses may indicate a risk that outbreaks are occurring in humans but not being detected, which could potentially happen in the future in humans. Serological surveillance in equines would only be feasible in animals not recently vaccinated or that have acquired natural immunity from prior virus exposure [53]. Finding seronegative equines in high-risk areas is expected to become increasingly difficult. Therefore, their role as risk indicators may be limited in areas with high virus exposure.

Further studies are needed to determine the extent to which equine data can be used as indicators of a prospective outbreak risk. Maintaining surveillance on horses is crucial, and it should be combined with surveillance in mosquitoes, birds and the human population to allow a more integrated and comprehensive approach in endemic countries [50], and promoting surveillance where necessary, particularly in countries with limited resources. Equine surveillance is more cost-effective than mosquito surveillance and more efficient than avian surveillance [50,54,55]. The role of horses as early indicators of risk should not be underestimated, as cases in horses may precede those in humans by several years. A strong link between equine and human alerts must exist to provide an effective and timely response and to enable the implementation of specific measures.

The role of river basins in outbreaks of WNV infection in the Iberian Peninsula is also noteworthy, as has been reported in other countries [33,56] such as in Italy in the river Po [57] and in Greece in the rivers Axios [58] and Strimon [59]. The areas of favourability for cases in humans and more markedly in horses, show a cartographic gradient corresponding to the basins of the Guadalquivir, Guadiana Tagus and the lower part of the Ebro, and the most favourable areas are closest to the coast. Water availability is higher in the lower areas of the basins, leading to higher vector densities. In the Iberian Peninsula, wetlands are situated in the lower part of the river basins, with a permanent presence of birds that significantly increases during the migration periods, mostly in summer and autumn (post-nuptial migration). In the Iberian Peninsula, outbreaks have historically been concentrated to the coastal area of the river Guadalquivir, in the areas of Seville and Cadiz. This would also explain the smaller numbers of cases and the less favourable conditions in the higher areas of these main river basins. The WNV risk model in horses not only predicted human cases of WNV infection in 2020, but also areas of previous outbreaks in equines and humans. All cases to date have occurred in areas highlighted by our model as high risk, developed from 2020 horse outbreak data. Moreover, all cases that were detected in 2021 and 2022 also occurred in the areas marked as at-risk by our model. All outbreaks in the Iberian Peninsula have occurred in the four highlighted main river basins, except for three outbreaks that occurred in other smaller basins, but always in the lower part of the basins.

## Conclusions

Based on our results, we propose river basins as-public health management units, rather than administrative units (such as municipalities or parishes). If cases are detected in horses, all health care centres within the same river basin should be alerted, as the spread can occur in neighbouring territories, even across countries like Portugal and Spain, if they share the same river basin. Decision-making would be more efficient from an ecosystem perspective, considering the environment and animal health as a whole, using in this case equines as identifiers of risk areas to ensure public health, i.e. the One Health approach. This biogeographic characterisation of the disease may help in the early identification of areas with a high potential risk, where cases in humans have not yet been detected. Our aim was to contribute to the knowledge of the viral ecology of this re-emerging pathogen in Europe and facilitate the development of surveillance programmes and preventive measures such as fumigation plans in sensitive areas or citizen awareness campaigns to reduce exposure and mosquito bites to reduce the impact of the disease and the number of people exposed to this virus.

## Supplementary Data

130000 Supplementary Material

## References

[1] Chancey C Grinev A Volkova E Rios M . The global ecology and epidemiology of West Nile virus. BioMed Res Int. 2015;2015:376230. 10.1155/2015/376230 25866777 PMC4383390

[2] Kramer LD Li J Shi PY . West Nile virus. Lancet Neurol. 2007;6(2):171-81. 10.1016/S1474-4422(07)70030-3 17239804

[3] Kaptoul D Viladrich PF Domingo C Niubó J Martínez-Yélamos S De Ory F West Nile virus in Spain: report of the first diagnosed case (in Spain) in a human with aseptic meningitis. Scand J Infect Dis. 2007;39(1):70-1. 10.1080/00365540600740553 17366016

[4] Connell J Mckeown P Garvey P Cotter S Conway A O’Flanagan D Two linked cases of West Nile virus (WNV) acquired by Irish tourists in the Algarve, Portugal. Euro Surveill. 2004;8(32):pii=2517. 10.2807/esw.08.32.02517-en

[5] Ministerio de Sanidad. Informe de Situación y Evaluación del Riesgo de la Fiebre por Virus del Nilo Occidental en España. [Information report and evaluation of the risk of West Nile fever in Spain]. Madrid: Ministerio de Sanidad; 2017. Spanish. Available from: https://www.sanidad.gob.es/profesionales/saludPublica/ccayes/analisisituacion/doc/Evaluacion_de_riesgo-VNO-2017.pdf

[6] García-Bocanegra I Jaén-Téllez JA Napp S Arenas-Montes A Fernández-Morente M Fernández-Molera V West Nile fever outbreak in horses and humans, Spain, 2010. Emerg Infect Dis. 2011;17(12):2397-9. 10.3201/eid1712.110651 22172565 PMC3311180

[7] Alves MJ Poças JMD Luz T Zé-Zé L Amaro F Osório H . Infecção por vírus West Nile (Flavivírus) em Portugal. Consideraçoes acerca de um caso clínico de síndrome febril. [West Nile virus (Flavivirus). infection in Portugal. Considerations about a clinical case with febrile syndrome and rash]. Rpdi. 2012; 8(1):46-51. Portuguese.

[8] García San Miguel Rodríguez-Alarcón L Fernández-Martínez B Sierra Moros MJ Vázquez A Julián Pachés P García Villacieros E Unprecedented increase of West Nile virus neuroinvasive disease, Spain, summer 2020. Euro Surveill. 2021;26(19):2002010. 10.2807/1560-7917.ES.2021.26.19.2002010 33988123 PMC8120797

[9] Lourenço J Barros SC Zé-Zé L Damineli DSC Giovanetti M Osório HC West Nile virus transmission potential in Portugal. Commun Biol. 2022;5(1):6. 10.1038/s42003-021-02969-3 35013546 PMC8748923

[10] Casimiro-Soriguer CS Perez-Florido J Fernandez-Rueda JL Pedrosa-Corral I Guillot-Sulay V Lorusso N Phylogenetic analysis of the 2020 West Nile virus (WNV) outbreak in Andalusia (Spain). Viruses. 2021;13(5):836. 10.3390/v13050836 34063166 PMC8148183

[11] Leblond A Hendrikx P Sabatier P . West Nile virus outbreak detection using syndromic monitoring in horses. Vector Borne Zoonotic Dis. 2007;7(3):403-10. 10.1089/vbz.2006.0593 17767410

[12] Ward MP Schuermann JA Highfield LD Murray KO . Characteristics of an outbreak of West Nile virus encephalomyelitis in a previously uninfected population of horses. Vet Microbiol. 2006;118(3-4):255-9. 10.1016/j.vetmic.2006.07.016 16971067

[13] García-Bocanegra I Belkhiria J Napp S Cano-Terriza D Jiménez-Ruiz S Martínez-López B . Epidemiology and spatio-temporal analysis of West Nile virus in horses in Spain between 2010 and 2016. Transbound Emerg Dis. 2018;65(2):567-77. 10.1111/tbed.12742 29034611

[14] van Galen G Calozet L Leblond A Tritz P Dal Pozzo F Porter SR Can horses be clinically screened for West Nile Fever? Vet Rec. 2013;172(4):101. 10.1136/vr.101267 23292842

[15] Saegerman C Alba-Casals A García-Bocanegra I Dal Pozzo F van Galen G . Clinical sentinel surveillance of equine West Nile fever, Spain. Transbound Emerg Dis. 2016;63(2):184-93. 10.1111/tbed.12243 24899369

[16] Barker CM Reisen WK Kramer VL . California state Mosquito-Borne Virus Surveillance and Response Plan: a retrospective evaluation using conditional simulations. Am J Trop Med Hyg. 2003;68(5):508-18. 10.4269/ajtmh.2003.68.508 12812335

[17] Reed KD Meece JK Henkel JS Shukla SK . Birds, migration and emerging zoonoses: west nile virus, lyme disease, influenza A and enteropathogens. Clin Med Res. 2003;1(1):5-12. 10.3121/cmr.1.1.5 15931279 PMC1069015

[18] Napp S Montalvo T Piñol-Baena C Gómez-Martín MB Nicolás-Francisco O Soler M Usefulness of Eurasian magpies (Pica pica) for West Nile virus surveillance in non-endemic and endemic situations. Viruses. 2019;11(8):716. 10.3390/v11080716 31387316 PMC6722797

[19] European Commission (EC). Commission implementing decision 2018/945 of 22 June 2018 on the communicable diseases and related special health issues to be covered by epidemiological surveillance as well as relevant case definitions. Brussels: EC; 2018. Available from: https://eur-lex.europa.eu/legal-content/EN/TXT/PDF/?uri=CELEX:32018D0945

[20] European Centre for Disease Prevention and Control (ECDC). West Nile virus transmission season in Europe, 2020. Stockholm: ECDC; 2021. Available from: https://www.ecdc.europa.eu/en/news-events/epidemiological-update-west-nile-virus-transmission-season-europe-2020

[21] Eurostat. NUTS- Nomenclature of Territorial Units for Statistics. Luxemburg: Eurostat; 2021. Available from: https://ec.europa.eu/eurostat/web/nuts/background

[22] Ministerio de Sanidad. Meningoencefalitis por el virus del Nilo Occidental en España. Evaluación rápida de Riesgo. [West Nile virus meningoencephalitis. Rapid risk assessment]. Madrid: Ministerio de Sanidad; 2020. Spanish. Available from: https://www.sanidad.gob.es/profesionales/saludPublica/ccayes/alertasActual/docs/20200925_ERR_Nilo_Occidental.pdf

[23] Ministerio de Sanidad. Meningoencefalitis por virus del Nilo occidental en España (2^a^ actualización). Evaluación rápida de riesgo. [West Nile virus meningoencephalitis in Spain (2nd update). Rapid risk assessment]. Madrid: Ministerio de Sanidad; 2020. Spanish. Available from: https://www.sanidad.gob.es/profesionales/saludPublica/ccayes/alertasActual/docs/20201203_ERR_Nilo_Occidental.pdf

[24] European Commission (EC). Animal Disease Information System. Brussels: EC. [Accessed: 28 Aug 2023]. Available from: https://food.ec.europa.eu/animals/animal-diseases/animal-disease-information-system-adis_en

[25] Benjamini Y Hochberg Y . Controlling the false discovery rate: a practical and powerful approach to multiple testing. J R Stat Soc. 1995;57(1):289-300.

[26] Rao CR . Large sample tests of statistical hypotheses concerning several parameters with applications to problems of estimation. Math Proc Camb Philos Soc. 1948;44(1):50-7. 10.1017/S0305004100023987

[27] Real R Barbosa AM Vargas JM . Obtaining environmental favourability functions from logistic regression. Environ Ecol Stat. 2006;13(2):237-45. 10.1007/s10651-005-0003-3

[28] Fielding AH Bell JF . A review of methods for the assessment of prediction errors in conservation presence/absence models. Environ Conserv. 1997;24(1):38-49. 10.1017/S0376892997000088

[29] Márcia Barbosa A Real R Muñoz A-R Brown JA . New measures for assessing model equilibrium and prediction mismatch in species distribution models. Divers Distrib. 2013;19(10):1333-8. 10.1111/ddi.12100

[30] Lobo JM Jiménez-Valverde A Real R . AUC: A misleading measure of the performance of predictive distribution models. Glob Ecol Biogeogr. 2008;17(2):145-51. 10.1111/j.1466-8238.2007.00358.x

[31] Bombi P D’Amen M . Scaling down distribution maps from atlas data: A test of different approaches with virtual species. J Biogeogr. 2012;39(4):640-51. 10.1111/j.1365-2699.2011.02627.x

[32] Bakonyi T Haussig JM . West Nile virus keeps on moving up in Europe. Euro Surveill. 2020;25(46):2001938. 10.2807/1560-7917.ES.2020.25.46.2001938 33213684 PMC7678036

[33] García-Carrasco J-M Muñoz A-R Olivero J Segura M Real R . Predicting the spatio-temporal spread of West Nile virus in Europe. PLoS Negl Trop Dis. 2021;15(1):e0009022. 10.1371/journal.pntd.0009022 33411739 PMC7790247

[34] Camp JV Nowotny N . The knowns and unknowns of West Nile virus in Europe: what did we learn from the 2018 outbreak? Expert Rev Anti Infect Ther. 2020;18(2):145-54. 10.1080/14787210.2020.1713751 31914833

[35] Bravo-Barriga D Aguilera-Sepúlveda P Guerrero-Carvajal F Llorente F Reina D Pérez-Martín JE West Nile and Usutu virus infections in wild birds admitted to rehabilitation centres in Extremadura, western Spain, 2017-2019. Vet Microbiol. 2021;255(March):109020. 10.1016/j.vetmic.2021.109020 33677369

[36] García-Bocanegra I Franco JJ León CI Barbero-Moyano J García-Miña MV Fernández-Molera V High exposure of West Nile virus in equid and wild bird populations in Spain following the epidemic outbreak in 2020. Transbound Emerg Dis. 2022;69(6):3624-36. 10.1111/tbed.14733 36222172 PMC10092718

[37] Ulbert S . West Nile virus vaccines - current situation and future directions. Hum Vaccin Immunother. 2019;15(10):2337-42. 10.1080/21645515.2019.1621149 31116691 PMC6816401

[38] Marini G Poletti P Giacobini M Pugliese A Merler S Rosà R . The role of climatic and density dependent factors in shaping mosquito population dynamics: The case of Culex pipiens in northwestern Italy. PLoS One. 2016;11(4):e0154018. 10.1371/journal.pone.0154018 27105065 PMC4841511

[39] Vogels CBF Hartemink N Koenraadt CJM . Modelling West Nile virus transmission risk in Europe: effect of temperature and mosquito biotypes on the basic reproduction number. Sci Rep. 2017;7(1):5022. 10.1038/s41598-017-05185-4 28694450 PMC5504010

[40] Brugueras S Fernández-Martínez B Martínez-de la Puente J Figuerola J Porro TM Rius C Environmental drivers, climate change and emergent diseases transmitted by mosquitoes and their vectors in southern Europe: A systematic review. Environ Res. 2020;191:110038. 10.1016/j.envres.2020.110038 32810503

[41] Reisen WK Fang Y Martinez VM . Effects of temperature on the transmission of west nile virus by Culex tarsalis (Diptera: Culicidae). J Med Entomol. 2006;43(2):309-17. 10.1093/jmedent/43.2.309 16619616

[42] Hartley DM Barker CM Le Menach A Niu T Gaff HD Reisen WK . Effects of temperature on emergence and seasonality of West Nile virus in California. Am J Trop Med Hyg. 2012;86(5):884-94. 10.4269/ajtmh.2012.11-0342 22556092 PMC3335698

[43] Paz S Malkinson D Green MS Tsioni G Papa A Danis K Permissive summer temperatures of the 2010 European West Nile fever upsurge. PLoS One. 2013;8(2):e56398. 10.1371/journal.pone.0056398 23431374 PMC3576399

[44] Caminade C Medlock JM Ducheyne E McIntyre KM Leach S Baylis M Suitability of European climate for the Asian tiger mosquito Aedes albopictus: recent trends and future scenarios. J R Soc Interface. 2012;9(75):2708-17. 10.1098/rsif.2012.0138 22535696 PMC3427500

[45] Miramontes R Jr Lafferty WE Lind BK Oberle MW . Is agricultural activity linked to the incidence of human West Nile virus? Am J Prev Med. 2006;30(2):160-3. 10.1016/j.amepre.2005.10.008 16459215

[46] Crowder DW Dykstra EA Brauner JM Duffy A Reed C Martin E West nile virus prevalence across landscapes is mediated by local effects of agriculture on vector and host communities. PLoS One. 2013;8(1):e55006. 10.1371/journal.pone.0055006 23383032 PMC3559328

[47] Hardy JL Houk EJ Kramer LD Reeves WC . Intrinsic factors affecting vector competence of mosquitoes for arboviruses. Annu Rev Entomol. 1983;28(1):229-62. 10.1146/annurev.en.28.010183.001305 6131642

[48] Liang G Gao X Gould EA . Factors responsible for the emergence of arboviruses; strategies, challenges and limitations for their control. Emerg Microbes Infect. 2015;4(3):e18. 10.1038/emi.2015.18 26038768 PMC4395659

[49] World Health Organization (WHO). West Nile virus. Geneva: WHO; 2017. Available from: https://www.who.int/news-room/fact-sheets/detail/west-nile-virus

[50] Young JJ Coulombier D Domanović D Zeller H Gossner CM European Union West Nile fever working group . One Health approach for West Nile virus surveillance in the European Union: relevance of equine data for blood safety. Euro Surveill. 2019;24(16):1800349. 10.2807/1560-7917.ES.2019.24.16.1800349 31014416 PMC6826348

[51] Ajuntament de Reus. Detectat un cas positiu de febre del Nil Occidental a Reus. [A positive case of West Nile fever has been detected in Reus]. Reus: Ajuntament de Reus; 2022. Spanish. Available from: https://www.reus.cat/noticia/detectat-un-cas-positiu-de-febre-del-nil-occidental-reus

[52] Ziegler U Lühken R Keller M Cadar D van der Grinten E Michel F West Nile virus epizootic in Germany, 2018. Antiviral Res. 2019;162(162):39-43. 10.1016/j.antiviral.2018.12.005 30550796

[53] Ministerio de Agricultura Pesca y Alimentación. Programa de vigilancia fiebre del Nilo Occidental 2022. [West Nile Fever Surveillance Program 2022]. Madrid: Ministerio de Agricultura Pesca y Alimentación; 2021. Spanish. Available from: https://www.mapa.gob.es/es/ganaderia/temas/sanidad-animal-higiene-ganadera/sanidad-animal/enfermedades/fiebre-nilo-occidental/F_O_Nilo.aspx

[54] Healy JM Reisen WK Kramer VL Fischer M Lindsey NP Nasci RS Comparison of the efficiency and cost of West Nile virus surveillance methods in California. Vector Borne Zoonotic Dis. 2015;15(2):147-55. 10.1089/vbz.2014.1689 25700046 PMC4340646

[55] Chevalier V Lecollinet S Durand B . West Nile virus in Europe: a comparison of surveillance system designs in a changing epidemiological context. Vector Borne Zoonotic Dis. 2011;11(8):1085-91. 10.1089/vbz.2010.0234 21548765

[56] García-Carrasco J-M Muñoz A-R Real R . Anticipating the locations in Europe of high-risk areas for West Nile virus outbreaks in 2021. Zoonoses Public Health. 2021;68(8):982-6. 10.1111/zph.12877 34242480

[57] Riccò M Peruzzi S Balzarini F . Epidemiology of West Nile virus infections in humans, Italy, 2012-2020: a summary of available evidences. Trop Med Infect Dis. 2021;6(2):61. 10.3390/tropicalmed6020061 33923347 PMC8167603

[58] Papa A Danis K Baka A Bakas A Dougas G Lytras T Ongoing outbreak of West Nile virus infections in humans in Greece, July-August 2010. Euro Surveill. 2010;15(34):1-9644. 10.2807/ese.15.34.19644-en 20807489

[59] Papa A Tsioka K Gewehr S Kalaitzopouou S Pervanidou D Vakali A West Nile fever upsurge in a Greek regional unit, 2020. Acta Trop. 2021;221:106010. 10.1016/j.actatropica.2021.106010 34129841

