# Supplementary material for: West Nile virus in the Iberian Peninsula: using equine cases to identify high-risk areas for humans
Source: Euro Surveill. 2023 Oct 5;28(40):2200844. doi: 10.2807/1560-7917.ES.2023.28.40.2200844 (PMC10557382; doi:10.2807/1560-7917.ES.2023.28.40.2200844)
Supplement: Supplementary Material [file 22-00844_GARCIA-CARRASCO_Supplement.pdf]

Supplementary Material

This supplementary material is hosted by *Eurosurveillance* as supporting information alongside the article “West Nile virus in the Iberian Peninsula: using equine cases to identify high-risk areas for humans” on behalf of the authors who remain responsible for the accuracy and appropriateness of the content. The same standards for ethics, copyright, attributions and permissions as for the article apply. Supplements are not edited by Eurosurveillance and the journal is not responsible for the maintenance of any links or email addresses provided therein.

Table S1. Explanatory variables used in the Iberian risk model.

Type	Subtype	Name	References
Human	Human concentration	Population density	[1]
		Distance to population center	[2]
	Livestock	Poultry density	[3]
		Farmed duck density	[3]
		Horse density	[3]
		Pig density	[3]
	Infrastructure	Distance to roads	[4]
		Continuous urban fabric	[5]
		Discontinuous urban fabric	[5]
		Industrial or commercial units	[5]
		Construction sites	[5]
		Road and rail networks and associated land	[5]
		Green urban areas	[5]
	Agriculture	Non-irrigated arable land	[5]
		Vineyards	[5]
		Olive groves	[5]
		Annual crops associated with permanent crops	[5]
		Rice fields	[5]
		Permanently irrigated land	[5]
		Fruit trees and berry plantations	[5]
		Pastures	[5]
		Complex cultivation patterns	[5]
		Land principally occupied by agriculture with significant areas of natural vegetation	[5]
		Agro-forestry areas	[5]
		% areas equipped with irrigation systems	[6]
Non-human	Ecosystem	Broad-leaved forest	[5]
		Coniferous forest	[5]
		Mixed forest	[5]
		Natural grasslands	[5]
		Moors and heathland	[5]
		Sclerophyllous vegetation	[5]
		Transitional woodland-shrub	[5]
		Sparsely vegetated areas	[5]
		Inland marshes	[5]
		Peat bogs	[5]
	Hydrographic	Salt marshes	[5]
		Salines	[5]
		Intertidal flats	[5]
		Water courses	[5]
		Water bodies	[5]
		Coastal lagoons	[5]
		Estuaries	[5]
		Distance to rivers	[7]
	Topographic	Altitude	[8]
		Slope	[9]
	Climatic	Annual Mean Precipitation	[10]
		Annual Mean Temperature	[10]
		Mean Temperature of Coldest Month	[10]
		Mean Temperature of the Hottest Month	[10]
		Max Temperature of the Hottest Month	[10]
		Min Temperature of the Coldest Month	[10]
		Coefficient of Variation of Precipitation	[10]
		Range of Temperature	[10]
		Surface Incoming Solar Radiation	[10]
		Surface Direct Irradiance	[10]

[1] LandScanTM 2008 High Resolution Global Population Data Set (copyrighted by UT-Battelle, LLC, operator of Oak Ridge National Laboratory), excluding any areas less than 2-km far from urban areas (as delimited by the MODIS 500 -m Map of Global Urban Extent for 2001-2002 (Schneider et al. 2009; 2010).
[2] Administrative Centres & Populated Places shapefile at the Relational World Database II (RWDB2) updated in 2000 (http://www.fao.org/geonetwork).
[3] Global FAO 2010 livestock (http://www.fao.org/livestock-systems/en/).
[4] Vector Map Level 0 at the Digital Chart of the World (DCW, http://worldmap.harvard.edu), updated in 2002.
[5] Corine Land Cover 2018 (https://land.copernicus.eu/pan-european/corine-land-cover/clc2018).
[6] Global Map of Irrigation Areas (version 4.0.1) around the year 2000
[7] Global Drainage Basin Database GDBD. Released Version 1.0: May 29, 2007 (http://www.cger.nies.go.jp/db/gdbd/gdbd_index_e.html).
[8] GTOPO30 (US Geological Survey 1996).
[9] Elaborated from DEM (Digital Elevation Model) using the altitude variable (GTOPO30; US Geological Survey 1996), using the Geographic Information Sistem ArcGIS Desktop 10.3.
[10] Agencia Estatal de Meteorología, Ministerio de Medio Ambiente y Medio Rural y Marino & Department of Meteorology and Climatology of the Institute of Meteorology, Portugal) (2011) Atlas climático ibérico: Temperatura del aire y precipitación (1971–2000). http://www.aemet.es
